# Effect of Resin Cement Color on the Final Color of Lithium Disilicate All-Ceramic Restorations

**Published:** 2018-05

**Authors:** Fariborz Vafaee, Bijan Heidari, Masoumeh Khoshhal, Amirarsalan Hooshyarfard, Mahmoud Izadi, Armaghan Shahbazi, Abbas Moghimbeigi

**Affiliations:** 1 Associate Professor, Implant Research Center, Department of Prosthodontics, School of Dental Medicine, Hamadan University of Medical Sciences, Hamadan, Iran; 2 Assistant Professor, Implant Research Center, Department of Prosthodontics, School of Dental Medicine, Hamadan University of Medical Sciences, Hamadan, Iran; 3 Assistant Professor, Implant Research Center, Department of Periodontics, School of Dental Medicine, Hamadan University of Medical Sciences, Hamadan, Iran; 4 Postgraduate Student, Department of Periodontics, School of Dental Medicine, Hamadan University of Medical Sciences, Hamadan, Iran; 5 Assistant Professor, Department of Prosthodontics, School of Dental Medicine, Ahvaz Jundishapur University of Medical Sciences, Ahvaz, Iran; 6 Postgraduate Student, Department of Prosthodontics, School of Dental Medicine, Hamadan University of Medical Sciences, Hamadan, Iran; 7 Professor, Modeling of Noncomunicable Disease Research Center, Department of Biostatistics, Faculty of Public Health, Hamadan University of Medical Sciences, Hamadan, Iran

**Keywords:** Resin Cement, Color, Variolink II, IPS e.max Press

## Abstract

**Objectives:**

Obtaining an adequate ceramic thickness to mask the substructure color is not always feasible, and appropriate use of a cement may be the only solution. This study aimed to evaluate the effect of the color of Variolink II resin cement on the final color of lithium disilicate glass ceramic restorations.

**Materials and Methods:**

In this in-vitro study, 90 discs of IPS e.max Press ceramic were evaluated. The ceramic discs were cemented to composite and amalgam blocks. The effect of the cement color and substructure on the final color of ceramic was analyzed by calculating the color change (ΔE) value using a spectrophotometer. Data were analyzed via three-way analysis of variance (ANOVA) and Tukey’s test.

**Results:**

The cement color had a statistically significant effect on the final color of ceramic (P≤0.001). The white, yellow, and translucent cements caused the highest color change (ΔE=4.558, 3.308, and 2.649, respectively). The effect of composite substructure and the yellow cement on the final color was less prominent compared to other combinations of cement and substructure (ΔE=2.043). The white cement over amalgam substructure showed the greatest effect on the final color (ΔE=4.890). The ΔE in HO group was less than that of other combinations (P<0.05), and the greatest ΔE was reported in MO group with the white cement (ΔE=6.255).

**Conclusions:**

The final color of the restoration is influenced by the cement color. Therefore, when IPS e.max Press is used over a metal core, it is recommended to use a cement with an HO ceramic.

## INTRODUCTION

Optimal color match of restorations with adjacent natural teeth is a major challenge in dentistry [1,2]. All-ceramic restorations (with no metal substructure) have a higher translucency; thus, they are suitable for use in the esthetic region [3,4]. Among different ceramic systems, lithium disilicate glass ceramics are highly popular due to their adequate strength (350–450 MPa), optimal bond to dental structures, easy fabrication process (the lost wax technique in comparison with the layering technique), and excellent esthetic properties [5,6]. Previous studies suggest that the thickness of ceramic should be at least 2 mm in order to mask the effect of the underlying discolored tooth or the abutment color on the final color of the restoration [7–9]. In many clinical cases, achieving a 2-mm axial reduction is not possible without encroaching the pulp and compromising the strength of the remaining tooth structure. In such cases, over-milling is often performed, which may compromise the durability of the restoration [10]. When achieving an optimal ceramic thickness is not feasible, using a cement with an appropriate thickness and color might be the only available solution to mask the color of the substructure and its effect on the final color of the restoration [10–16].

Spectrophotometers are among the most accurate tools for tooth color measurement in dentistry [17–19]. The color change (ΔE) value is often used to compare two different colors and is calculated using the following equation: ΔE=(ΔL2 + Δa2 + Δb2)½. If ΔE is bigger than one, the color difference is significant and visible for at least 50% of the observers. In case of ΔE>2.7, the color difference is not clinically acceptable [20].

The effect of the resin cement color on the final color of ceramic restorations has been previously evaluated; however, no applicable guideline is available regarding the use of different cement colors in the clinical setting [11,12,14,15]. Therefore, considering the introduction of new ceramics with different translucencies, more studies are required to understand the effect of the substructure and the cement color on the final color of ceramic.

The aim of the current study was to evaluate the effect of the color of Variolink II resin cement on the final color of IPS e.max Press ceramics to obtain the best combination in use of all-ceramic restorations in terms of the color match.

## MATERIALS AND METHODS

This in-vitro experimental study evaluated the effect of the cement color on the final color of lithium disilicate glass ceramics. Ninety specimens were fabricated for the assessment of high opacity (HO), medium opacity (MO), and low translucency (LT) cores, three cement colors (white, yellow, and transparent), and two types of substructure (amalgam and composite). The specimens were randomly divided into 18 groups of five using a table of random numbers. The MO and HO cores were fabricated as double-layer (along with a veneering layer) according to the manufacturer’s instructions, while the LT core was fabricated in the form of monolayer without veneering. For the fabrication of lithium disilicate ceramic cores (A2 shade of IPS e.max Press; Ivoclar Vivadent, Schaan, Liechtenstein) with the thickness of 1.2 mm and the diameter of 12 mm, the lost wax technique was used and investment was performed according to the manufacturer’s instructions.

**Fig. 1:**
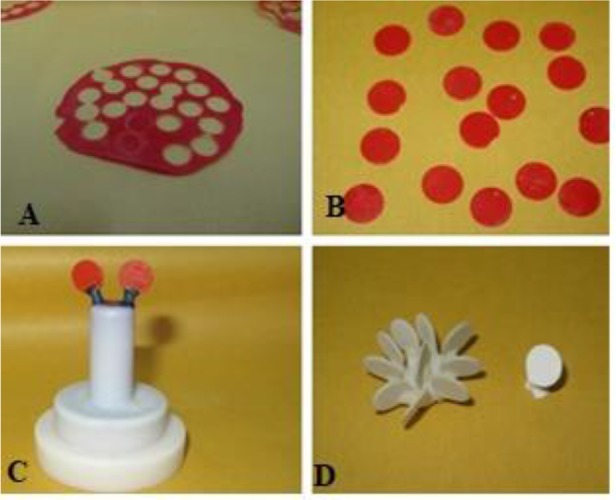
(A) Fabrication of acrylic patterns. (B) Cutting out the discs from the acrylic sheet. (C) Spruing the discs. (D) Separating the discs from the sprue

For this purpose, wax and acrylic patterns with the shape and size of the desired discs were required. These discs had to be 12 mm in diameter and 0.6 mm in thickness (according to the manufacturer’s instructions regarding the minimum required thickness). For the fabrication of these acrylic patterns, four metal stops measuring 3×3 mm with the thickness of 0.6 mm were fabricated to be placed between two glass slabs creating a 0.6-mm space. Next, according to the manufacturer’s recommendations, a paste was prepared by mixing the monomer liquid and polymer powder (Pattern Resin; GC. Corp., Tokyo, Japan), which was placed between the two glass slabs. After complete setting, a 0.6-mm thick acrylic sheet was fabricated. For the fabrication of monolayer LT discs with the thickness of 1.2 mm, stops with the same thickness were used. Next, the discs with the same diameter were cut out of the acrylic sheet using a trephine bur (Biomet 3i, Palm Beach, FL, USA; Fig. 1). These discs were sprued and used as a pattern for the fabrication of ceramic cores. After casting, the discs were separated from the sprue using a separating disc (Ivoclar Vivadent, Schaan, Liechtenstein) and were then ground using the grinder discs recommended by the manufacturer. The thickness of the discs was adjusted using a digital caliper (Maxwell Digital Caliper, Ohio, USA). These stages were also performed for HO and MO cores. For the LT ceramic, the same procedures were followed to fabricate a 1.2-mm thick pattern to obtain monolayer LT ceramic discs. Next, in order to add a 0.6-mm thick veneering layer over the core, a mold was used, which was made of a metal sheet measuring 30×30 mm with the thickness of 1.2 mm. There was a 13-mm-diameter hole at the center of the mold such that the core discs could be easily placed in it. The cores were placed in the hole, and the IPS e.max Ceram layering (veneering) was applied. Excess material was carved. To compensate for porcelain shrinkage, the porcelain was applied over each disc twice and was baked to obtain a 1.2-mm thickness. The discs were then finished using Diagen-Turbo-Grinder discs (Ivoclar Vivadent, Schaan, Liechtenstein) to create an equal thickness. A round-end cylindrical bur (S835R/012, SwissTEC, Switzerland) was used for creating a groove measuring 3×1.2 mm, perpendicular to the periphery of the disc, and then, all double-layer MO and HO and monolayer LT discs were auto-glazed according to the manufacturer’s instructions. In this study, amalgam (Sinaluxe, Shahid Faghihi Co., Alborz, Iran) and composite (Tetric Ceram®, Ivoclar Vivadent, Schaan, Liechtenstein) substructures were fabricated as follows: a metal disc with the thickness of 1.3 mm and the diameter of 13 mm with a groove perpendicular to the periphery (measuring 2.5×1 mm) was glued to the bottom of a plastic container (at the center) measuring 30×30 mm with a 5-mm depth using liquid glue. A paper disc with the diameter of 10 mm and the thickness of 30μm was glued to the metal disc (at the center). The amalgam was then condensed around the metal disc as recommended by the manufacturer until the plastic container was filled with amalgam. Similarly, the composite was incrementally applied to the plastic container around the disc such that the container was filled with composite. After complete setting, the substructures were removed from the container.

**Fig. 2:**
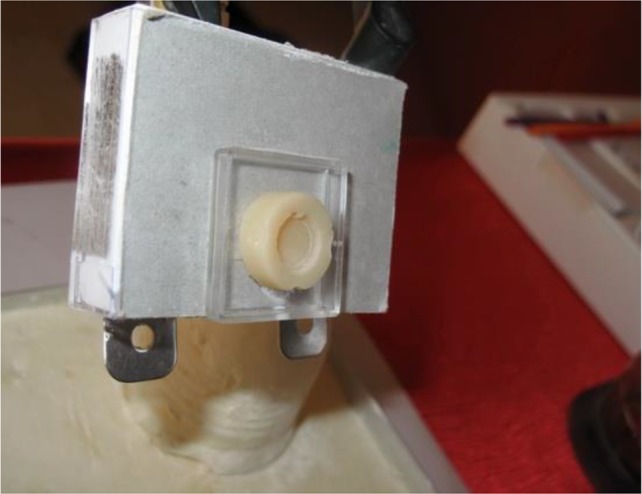
Fixing the composite block using a custom-made fixing tool

**Fig. 3:**
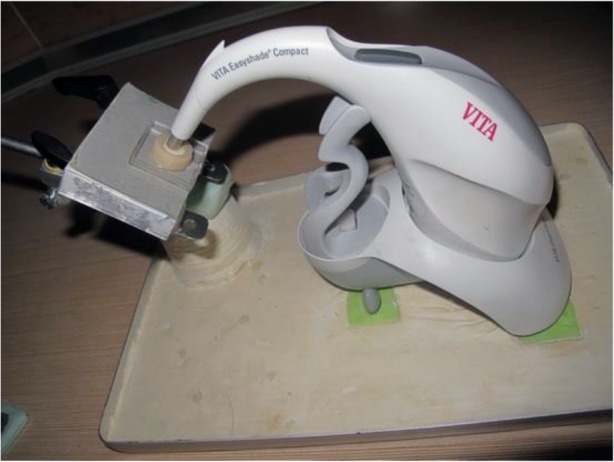
Fixing the disc in front of the spectrophotometer by the fixing tool

The presence of a metal disc created a space inside the composite and amalgam for subsequent placement of ceramic discs. The paper disc on top of the substructures created a 30-μm space for the cement. The groove at the peripheral margin of the metal disc created an appendage in composite and amalgam blocks to match the groove in the ceramic disc. This was done for reproducible positioning of ceramic discs. Also, the same area of the discs was subjected to spectrophotometry (Fig. 2). Variolink II resin cement (Ivoclar Vivadent, Schaan, Liechtenstein) was used for cementation. The ceramic discs were cemented to composite and amalgam blocks. The effect of the color of amalgam and composite substructures and the cement color on IPS e.max Press ceramic was compared with that of the control group (A2 shade of ceramic, glycerin instead of cement) using a VITA Easyshade spectrophotometer (Vita Zahnfabrik, Bad Säckingen, Germany). In order to fix the disc in front of the spectrophotometer, a fixing tool was needed, which was designed by fixing a mounting jig base of a Bio-art articulator (Bio-Art, São Carlos, Brazil) in stone plaster type III (GC America Inc., Alsip, IL, USA) which was placed in a dental aluminum tray. Amalgam and composite substructures were attached to the fork of the spectrophotometer using a plastic plate (Fig. 3). The color change (ΔE) of the test groups and the control group was calculated according to the following formula: ΔE=(ΔL2 + Δa2 + Δb2)½, where ΔE is the color change, ΔL refers to change in lightness (L parameter), Δa refers to change in a* color parameter (indicative of greenness-redness), and Δb refers to change in b* color parameter (indicative of blueness-yellowness).

Data were collected and analyzed by SPSS version 18 software (SPSS Inc., Chicago, IL, USA) via three-way analysis of variance (ANOVA) and Tukey’s test with the level of significance set at 0.05.

## RESULTS

Three-way ANOVA was used to assess the effect of the study variables on the final color of the restoration. Table 1 presents the results of this test. As shown, the interaction effect of the substructure, cement, and ceramic on the final color of the restoration was not significant (P=0.217); however, other interactions were significant (P<0.05).

**Table 1.** Effect of the cement color on the final color of ceramic according to the type of the substructure and ceramic (three-way ANOVA)

**Source of variation**	**Type III sum of squares**	**Df**	**Mean square**	**F distribution**	**Sig.**
Substructure	10.804	1	10.804	4.611	0.035
Cement	56.375	2	28.188	12.030	0.000
Ceramic	129.722	2	64.861	27.681	0.000
Substructure * cement	40.870	2	20.435	8.721	0.000
Substructure * ceramic	18.601	2	9.300	3.969	0.023
Cement * ceramic	52.544	4	13.136	5.606	0.001
Substructure * cement * ceramic	13.890	4	3.472	1.482	0.217
Error	168.709	72	2.343		
Total	1597.300	90			
Corrected Total	491.515	89			

* Interaction, Df=Degree of freedom

The results showed that the mean ΔE for the three ceramic groups after cementation with three different cement colors on two different substructures was 1.807, 4.353, and 4.355 for HO, MO, and LT ceramics, respectively. Since the effect of different cement colors on the final color of the restoration was significant (P<0.001), Tukey’s test was used for pairwise comparisons of the effect of the cements. Table 2 shows the results of Tukey’s test. The mean ΔE for the three groups of white, yellow and transparent cements after cementation of different ceramics on the substructure was found to be 4.558, 3.308, and 2.649, respectively.

**Table 2.** Multiple Comparisons of the mean ΔE in three different cement groups

**Cement**		**(I-J=) ΔE MD**	**P-Value**

**I (Group one)**	**J (Group two)**		
White	Transparent	(4.558–2.649=) 1.909	<0.001
	Yellow	(4.558–3.308=) 1.250	<0.001
Yellow	Transparent	(3.308–2.649=) 0.659	0.018

MD=Mean Difference

Considering that the interaction effect of the substructure and the cement on the final color of the restoration was found to be significant (P<0.001), Tukey’s test was applied for pairwise comparisons (Table 3). As shown in Table 3, the mean ΔE for the combination of composite substructure and the yellow cement was less than that of other combinations (ΔE=2.043). The application of the white cement along with amalgam substructure yielded the highest ΔE (4.890). Based on the results of this study, the interaction effect of the cement and ceramic on the final color of the restoration was significant (P=0.001); therefore, Tukey’s test was applied (Table 4). As shown in Table 4, the ΔE after cementation in HO group was less than that of other combinations (P<0.001), and the greatest ΔE was reported in MO group with the white cement (ΔE=6.255).

**Table 3.** Multiple comparisons of the mean ΔE in different combinations of substructure and cement

**Substructure**	**Cement**		**(I-J=) ΔE MD**	**P-Value**

	**I (Group one)**	**J (Group two)**		
**Amalgam**	White	Transparent	(4.890–2.756=) 2.134	<0.001

		Yellow	(4.890–4.574=) 0.316	0.423

	Yellow	Transparent	(4.574–2.756=) 1.818	<0.001
**Composite**	White	Yellow	(4.225–2.043=) 2.182	<0.001

		Transparent	(4.225–2.543=) 1.682	<0.001

	Transparent	Yellow	(2.543–2.043=) 0.500	0.208

MD=Mean Difference

**Table 4.** Multiple comparisons of the mean ΔE in different combinations of cement and ceramic

**Cement**	**Ceramic**		**(I-J=) ΔE MD**	**P-Value**

	**I (Group one)**	**J (Group two)**		
**Yellow**	**LT**	HO	(4.038–2.247=) 1.791	<0.001
		MO	(4.038–3.640=) 0.398	0.411
	**MO**	HO	(3.640–2.247=) 1.393	<0.001
**Transparent**	**MO**	HO	(3.163–1.820=) 1.343	<0.001
		LT	(3.163–2.965=) 0.198	0.682
	**LT**	HO	(2.965–1.820=) 1.145	<0.001
**White**	**MO**	HO	(6.255–1.355=) 4.900	<0.001
		LT	(6.255– 6.063=) 0.192	0.691
	**LT**	HO	(6.063–1.355=) 4.708	<0.001

MD=Mean Difference, HO=High Opacity, MO=Medium Opacity, LT=Low Translucency

## DISCUSSION

In order to fabricate an esthetic restoration, clinicians and technicians should take into account all the factors that affect the final color of the restoration. In the current study, the effect of the cement color on the final color of ceramic with metal and composite substructures was evaluated. The results of three-way ANOVA showed that the cement color had a significant effect on the final color of ceramic (P<0.001).

The effect of different IPS e.max Press ceramic ingots (HO, MO, and LT) on the final color of the restoration was also studied. Based on the manufacturer’s claim, an HO ceramic with the thickness of 1.2 mm is capable of masking the silver color of amalgam. In our study, the color of the MO ceramic was significantly influenced by the silver color of amalgam.

The results of our study indicated that the influence of the cement color on the final color of ceramic was significantly minimized by using HO ceramic discs (P<0.001). Considering the mean ΔE of the HO ceramic (ΔE=1.807), it can be stated that the cement color has a significant effect on the final color of the restoration recognizable by at least 50% of the observers while being clinically acceptable. On the other hand, the mean ΔE of the MO (ΔE=4.353) and LT (ΔE=4.355) ceramics indicated that the cement color had a significant effect on the final color of the restoration, bringing it closer to the clinically acceptable range.

The results of the current study showed that the final color of the restoration was significantly influenced by the cement color. Based on the ΔE value, the white and yellow cements used in this study can change the final color of ceramic so that it would be clinically acceptable. The white cement not only had a greater effect on the final color of ceramic but also decreased the effect of the substructure color on the final color of the restoration. Contrariwise, the translucent cement was the only studied cement that caused no clinical change in the restoration color compared to the color before cementation (ΔE≤2.7).

Chang et al [14] evaluated the effect of Variolink II, Esthetic, and Nexus II cements on the color of IPS Empress (Ivoclar) and Katana (Noritake) ceramic restorations. They found that the combination of the cement color with the substructure color and ceramic can influence the final color of the restoration [14].

The results of their study and ours are in contrast to those found by Terzioğlu et al [21] who used RelyX ARC (3M ESPE) cement in two colors and IPS Empress ceramic. They found that the ceramic color significantly changed after cementation, but no significant difference was noted between different cement colors [21].

Karaagaclioglu and Yilmaz [22] studied the effect of the cement color on the final color of ceramic; their results were not in accordance with the results of the current study. They used IPS Empress ceramic with the thickness of 0.8 mm and RelyX ARC cement in A1 and A3 shades. Their results did not show any significant difference between the effects of the two cement colors on the final color of the ceramic [22].

There are three factors that affect the final color of ceramic restorations: the first factor is the effect of the ceramic color. At present, various types of ceramics with highly different optical properties are available, and the type of the applied ceramic should be considered in the interpretation and comparison of the results. The second factor is the effect of cement polymerization on the final color of the restoration. Kucukesmen et al [23] reported that the curing process of the cement influences the final color. Therefore, the results of studies in which the cement is properly cured are scientifically more reliable since they better simulate the clinical setting [23]. The third factor is the difference between the color of the main cement and the try-in paste. Although the manufacturers produce try-in pastes that are compatible with the main cement, El-Hejazi and Alsufayyan [24] showed that the final color of the restoration changes after using the main cement instead of the try-in paste.

By comparing the mean value of ΔE associated with the combination of substructure and cement, it can be concluded that when using a composite substructure and a yellow cement (ΔE=2.043), the final color of ceramic is influenced less by the cement in comparison with other combinations. Also, this effect was most prominent when using the white cement and an amalgam substructure (ΔE=4.890). The comparison of the mean ΔE value in use of ceramic and cement showed that the mean ΔE of the white cement and the HO ceramic was the lowest compared to other combinations; whereas the greatest ΔE in use of the white cement (ΔE=6.255) and the translucent cement (ΔE=3.163) was related to the MO ceramic core. However, for the yellow cement (ΔE=4.038), the greatest ΔE was reported when an LT ceramic core was used.

## CONCLUSION

According to the results of the present study, we may conclude that when an MO ceramic core is used with a dental substrate or with a substructure of a suitable color, the color change resulted from the application of the cement is more prominent. In case of discoloration of the dental substrate or improper substructure color, the optimal final color should be achieved by choosing the proper cement color. It seems that the combination of a white cement and an HO ceramic core would be a proper combination to prevent the adverse effect of the substructure color on the final color of the restoration.
